# Predict, diagnose, and treat chronic kidney disease with machine learning: a systematic literature review

**DOI:** 10.1007/s40620-023-01573-4

**Published:** 2023-02-14

**Authors:** Francesco Sanmarchi, Claudio Fanconi, Davide Golinelli, Davide Gori, Tina Hernandez-Boussard, Angelo Capodici

**Affiliations:** 1 Department of Biomedical and Neuromotor Science, Alma Mater Studiorum, University of Bologna, Via San Giacomo 12, 40126 Bologna, Italy; 2 Department of Medicine (Biomedical Informatics), Stanford University, School of Medicine, Stanford, CA USA; 3 Department of Electrical Engineering and Information Technology, ETH Zurich, Zurich, Switzerland

**Keywords:** Chronic kidney disease, Machine learning, Artificial intelligence, Systematic review

## Abstract

**Objectives:**

In this systematic review we aimed at assessing how artificial intelligence (AI), including machine learning (ML) techniques have been deployed to predict, diagnose, and treat chronic kidney disease (CKD). We systematically reviewed the available evidence on these innovative techniques to improve CKD diagnosis and patient management.

**Methods:**

We included English language studies retrieved from PubMed. The review is therefore to be classified as a “rapid review”, since it includes one database only, and has language restrictions; the novelty and importance of the issue make missing relevant papers unlikely. We extracted 16 variables, including: main aim, studied population, data source, sample size, problem type (regression, classification), predictors used, and performance metrics. We followed the Preferred Reporting Items for Systematic Reviews (PRISMA) approach; all main steps were done in duplicate.

**Results:**

From a total of 648 studies initially retrieved, 68 articles met the inclusion criteria.

Models, as reported by authors, performed well, but the reported metrics were not homogeneous across articles and therefore direct comparison was not feasible. The most common aim was prediction of prognosis, followed by diagnosis of CKD. Algorithm generalizability, and testing on diverse populations was rarely taken into account. Furthermore, the clinical evaluation and validation of the models/algorithms was perused; only a fraction of the included studies, 6 out of 68, were performed in a clinical context.

**Conclusions:**

Machine learning is a promising tool for the prediction of risk, diagnosis, and therapy management for CKD patients. Nonetheless, future work is needed to address the interpretability, generalizability, and fairness of the models to ensure the safe application of such technologies in routine clinical practice.

**Graphical abstract:**

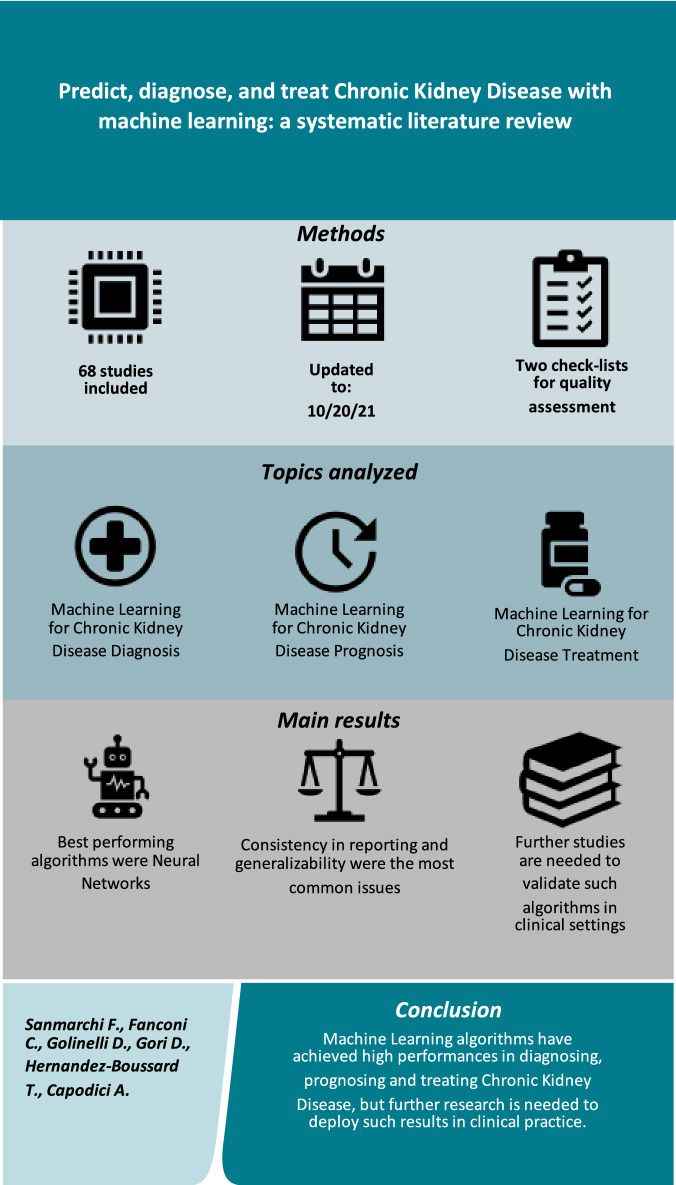

**Supplementary Information:**

The online version contains supplementary material available at 10.1007/s40620-023-01573-4.

## Introduction

Chronic Kidney Disease (CKD) is a state of progressive loss of kidney function ultimately resulting in the need for renal replacement therapy (dialysis or transplantation) [1]. It is defined as the presence of kidney damage or an estimated glomerular filtration rate less than 60 ml/min per 1.73 m^2^, persisting for 3 months or more [2]. CKD prevalence is growing worldwide, along with demographic and epidemiological transitions [3]. The implications of this disease are enormous for our society in terms of quality of life and the overall sustainability of national health systems. Worldwide, CKD accounted for 2,968,600 (1%) disability-adjusted life-years and 2,546,700 (1% to 3%) life-years lost in 2012 [4]. Therefore, it is of the utmost importance to assess how to promptly and adequately diagnose and treat patients with CKD.

The causes of CKD vary globally. The most common primary diseases causing CKD and ultimately kidney failure are diabetes mellitus, hypertension, and primary glomerulonephritis, representing 70–90% of the total primary causes [1, 2, 4]. Although these three causes are at the top of the CKD etiology charts, other features are involved in CKD pathophysiology (e.g., pollution, infections and autoimmune diseases) [5–9]. Similarly, there are numerous factors that play a role in CKD progression, namely non-modifiable risk factors (e.g., age, gender, ethnicity) and modifiable ones (e.g., systolic and diastolic blood pressure, proteinuria) [1, 2, 4–9].

Given how dauntingly vast the number of factors that can play a significant role in the etiology and progression of CKD is, it can be difficult to correctly assess the individual risk of CKD and its progression. Naturally, as with any complex problem, humans seek simplification, and therefore the question shifts to what to take into account when assessing CKD risk. Thanks to new methodological techniques, we now have the ability to improve our diagnostic and predictive capabilities.

Artificial Intelligence (AI) is the capacity of human-built machines to manifest complex decision-making or data analysis in a similar or augmented fashion in comparison to human intelligence [10]. Machine Learning (ML) is the collection of algorithms that empower models to learn from data, and therefore to undertake complex tasks through complex calculations [11–15]. In recent years AI and ML have offered enticing solutions to clinical problems, such as how to perform a diagnosis from sparse and seemingly contrasting data, or how to predict a prognosis [16]. Given the enormous potential of ML, and its capacity to learn from data, researchers have tried to apply its capacities to resolve complex problems, such as predicting CKD diagnosis and prognosis, and managing its treatment.

In this complex scenario, we aimed to systematically review the published studies that applied machine learning in the diagnosis and prediction, prognosis, and treatment of CKD patients. In doing so, the primary objective is to describe how ML models and variables have been used to predict, diagnose and treat CKD, as well as what results have been achieved in this field.

## Methods

### Search strategy and selection criteria

We conducted a systematic literature review, following the Preferred Reporting Items for Systematic Reviews (PRISMA) approach [17], including studies that applied ML algorithms to CKD forecasting, diagnosis, prognosis, and treatment. This systematic review’s outcomes of interest are machine learning models, features used, performances and uses regarding diagnosis, prognosis and treatment of CKD. The review itself and its protocol were not registered.

The initial search was implemented on October 20, 2021. The search query consisted of terms considered pertinent by the authors.

We searched for publications on PubMed using the following search string: “((artificial intelligence[Title/Abstract]) OR (machine learning[Title/Abstract]) OR (computational*[Title/Abstract]) OR (deep learning[Title/Abstract])) AND ((ckd) OR (chronic kidney disease) OR (chronic kidney injury) OR (chronic kidney) OR (chronic renal) OR (end stage renal) OR (end stage kidney) OR (ESKD) OR (ESRD) OR (CKJ) OR (CKI) OR (((renal) OR (kidney)) AND (failure)))”.

We included articles for review if they were in vivo studies (human-based), which applied AI & ML techniques in order to assess the diagnosis, prognosis, or therapy of CKD patients and reported original data. We did not limit our inclusion criteria to any specific study design, nor to any outcome of interest, as our main goal was to be as inclusive as possible, and we wanted to capture all available evidence from any study design and any outcome of interest.

We excluded studies that were not in English, those focusing on animals, reviews, systematic reviews, opinions, editorials, and case reports. We decided to exclude in vitro studies (conducted on cellular substrates) and studies focusing on animals, in order to summarize the current evidence on the application of ML models on humans.

### Data extraction

Data were extracted by two independent reviewers (AC and FS). Disagreement on extracted data was discussed with an independent arbiter (DGol).

The following data were extracted from each included article (main text and/or supplementary material): author(s) name, date of publication, first author affiliation (country and region), main study objective, objective category (risk, diagnosis, prognosis, and treatment), prognosis category, study population, data source, sample size, problem type (regression, classification), machine learning algorithms examined in the study, predictor categories, number of predictors used, predictor list, performance metrics, final conclusions, use in clinical context and the 5 most important model features. When more than one model was considered in the study, the one the authors deemed best was extracted. Performance metrics always refer to the models’ performance on test sets.

### Quality and risk assessment

Evaluation of the included studies was performed using both PROBAST [18] and the Guidelines for developing and reporting machine learning predictive models in biomedical research developed by Luo and colleagues [19].

## Results

### Included studies

**Fig. 1:**
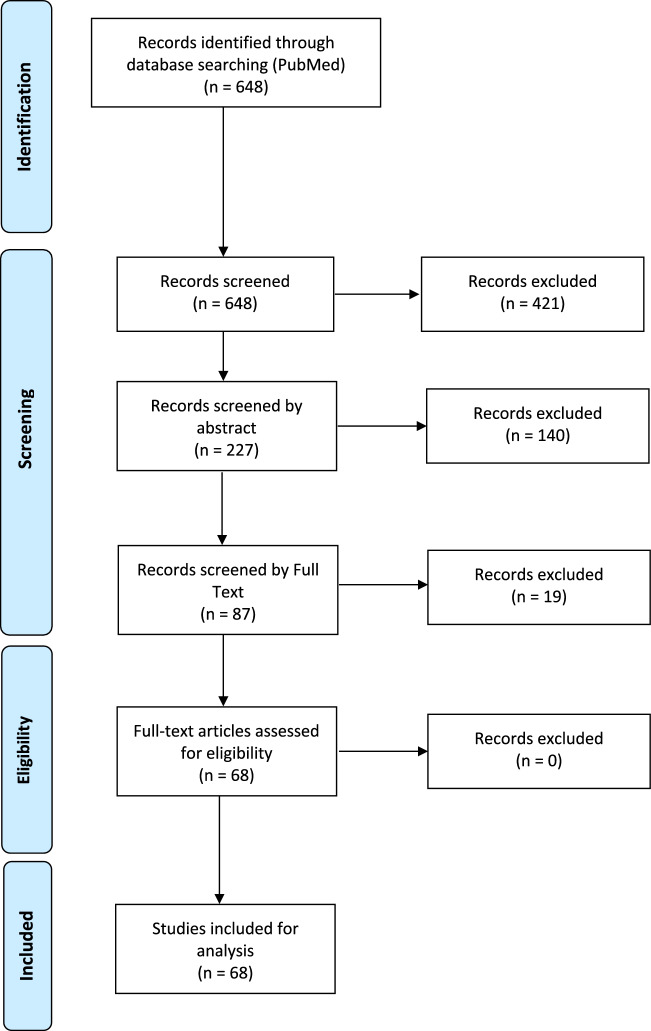
PRISMA flow-chart

Of the 648 articles retrieved from PubMed, 421 were ruled out after title screening, and 140 were excluded after abstract screening; a total of 87 articles were selected for full-text screening (Fig. 1). Of these 87 studies, 68 were included in the final set of articles (Table 1) [20–87].

**Table 1:** Extracts of the main findings

Authors	Country	Year	Sample size	Main Aim	Model task	Selected model	Performance metric(s)	Clinical Deployment
Akl et al	Africa	2001	30	Prognosis	Regression	ANN	–	No
Kusiak et al	North America	2005	188	Prognosis	Classification	Decision tree	Accuracy: 85	No
Chen et al	Asia	2007	153	Prognosis	Regression	ANN	–	No
Luo et al	North America	2013	66,633	Prognosis	Classification	Hidden Markov Model	–	No
Escandell-Montero et al	Europe	2014	128	Therapy	Regression	Markov decision processes	–	No
Martínez-Martínez et al	Europe	2014	13,011	Prognosis	Regression	Ensemble model	MSE: 0.90 MAE: 0.67	No
Barbieri et al	Europe	2015	4135	Prognosis	Regression	ANN	MSE: 0.75 MAE: 0.55	No
Singh et al	North America	2015	6435	Prognosis	Classification	ANN	AUC: 0.72 Sensitivity: 54	No
Barbieri et al	Europe	2016	752	Therapy	Regression	ANN	–	Yes
Chen et al	Asia	2016	400	Risk/forecast	Classification	Support vector machine	Accuracy: 99 Sensitivity: 100 Specificity: 99	No
Norouzi et al	Asia	2016	465	Diagnosis + Prognosis	Regression	ANN	MSE: 54.88 MAE: 5.50	No
Rodriguez et al	Europe	2016	1758	Prognosis	Regression	Random forest	–	No
Goldstein et al	North America	2017	18,846	Prognosis	Classification	LASSO regression	AUC: 0.84	No
Polat et al	Europe	2017	400	Diagnosis	Classification	Support vector machine	AUC: 0.99 Sensitivity: 98	No
Kleiman et al	North America	2018	401	Prognosis	Classification	Random forest	AUC: 0.86 Accuracy: 54	No
Kolachalama et al	North America	2018	171	Diagnosis	Classification	CNN	AUC: 0.91	No
Tang et al	Asia	2018	173	Risk/forecast	Classification	Random forest	–	No
Akbilgic et al	North America	2019	27,615	Prognosis	Classification	Random forest	AUC: 0.69	Yes
Almansour et al	Africa	2019	400	Diagnosis	Classification	ANN	Accuracy: 99 Sensitivity: 99 Specificity: 100	No
Elhoseny et al	Africa	2019	400	Diagnosis	Classification	ANN	Accuracy: 95 Sensitivity: 96 Specificity: 93	No
Forné et al	Europe	2019	1366	Prognosis	Classification	Random forest	AUC: 0.74	No
Galloway et al	North America	2019	449,380	Prognosis	Classification	CNN	AUC: 0.87	
Guo et al	South America	2019	703	Diagnosis + Prognosis	Classification	LASSO regression	Accuracy: 99	Yes
Han et al	Asia	2019	1370	Risk/forecast	Classification	Random forest	Accuracy: 93 Sensitivity: 80 Specificity: 95	No
Huang et al	Asia	2019	400	Diagnosis	Classification	ANN	Accuracy: 99 Sensitivity: 99 Specificity: 99	No
Kanda et al	Asia	2019	7465	Prognosis	Classification	Support vector machine	Accuracy: 89	No
Kannan et al	North America	2019	171	Diagnosis	Classification	CNN	Accuracy: 95 Sensitivity: 56 Specificity: 99	No
Kuo et al	Asia	2019	1299	Diagnosis	Classification	CNN	Accuracy: 86	No
Lin et al	Asia	2019	48,153	Prognosis	Classification	Random forest	MSE: 0.75 MAE: 0.51	No
Navaneeth et al	Asia	2019	104	Diagnosis	Classification	CNN	Accuracy: 98 Sensitivity: 98 Specificity: 98	No
Yu et al	North America	2019	703	Diagnosis	Classification	ANN	Accuracy: 99	No
Aldhyani et al	Africa	2020	768	Diagnosis	Classification	Support vector machine	Accuracy: 100 Sensitivity: 100 Specificity: 100	No
Belur Nagaraj et al	Europe	2020	11,789	Prognosis	Classification	ANN	AUC: 0.82	No
Chen et al	Asia	2020	101	Diagnosis	Classification	Support vector machine	Accuracy: 90 Sensitivity: 100 Specificity: 79	No
Dovgan et al	Europe	2020	8492	Prognosis	Classification	XGBoost	AUC: 0.78 Sensitivity: 62 Specificity: 78	No
Garcia-Montemayor et al	Europe	2020	1571	Prognosis	Classification	Random forest	AUC: 0.7 Accuracy: 73	No
Glazyrin et al	Asia	2020	48	Diagnosis	Classification	K nearest neighbor	Accuracy: 87	No
Huang et al	Europe	2020	3080	Risk/forecast	Classification	Random forest	AUC: 0.86	No
Inaguma et al	Asia	2020	19,894	Prognosis	Classification	Random forest	AUC: 0.73	No
Jeong et al	Asia	2020	134,895	Diagnosis	Classification	ANN	Accuracy: 99	No
Kanda et al	Asia	2020	79,860	Prognosis	Classification	Ensemble model	Accuracy: 95 Sensitivity: 91 Specificity: 99	Yes
Komaru et al	Asia	2020	101	Prognosis	Classification	Hierarchical clustering	AUC: 0.8	Yes
Kumar et al	Asia	2020	400	Diagnosis	Classification	Genetic algorithms	Accuracy: 99 Sensitivity: 99 Specificity: 100	No
Noh et al	Asia	2020	1730	Prognosis	Classification	ANN	AUC: 0.86	No
Nusinovici et al	Asia	2020	6762	Risk/forecast	Classification	Logistic regression	AUC: 0.90 Sensitivity: 80 Specificity: 60	No
Ogunleye et al	Africa	2020	400	Diagnosis	Classification	XGBoost	Accuracy: 100 Sensitivity: 100 Specificity: 100	No
Pellicer-Valero et al	Europe	2020	110,758	Prognosis	Regression	RNN	MSE: 0.72 MAE: 0.65	No
Roth et al	Europe	2020	12,761	Risk/forecast	Classification	RNN	AUC: 0.96	No
Sabanayagam et al	Asia	2020	5188	Diagnosis	Classification	ANN	AUC: 0.91	No
Segal et al	Asia	2020	550,000	Prognosis	Classification	XGBoost	AUC: 0.93 Sensitivity: 72 Specificity: 96	No
Shih et al	Asia	2020	19,270	Risk/forecast	Classification	Decision tree	AUC: 0.79 Accuracy: 82 Sensitivity: 67 Specificity: 79	No
Song et al	North America	2020	14,039	Risk/forecast	Classification	Gradient boosting machine	AUC: 0.83 Sensitivity: 83 Specificity: 78	No
Vitsios et al	Europe	2020	12,713	Risk/forecast	Classification	Random forest	AUC: 0.84	No
Weber et al	Europe	2020	785	Diagnosis	Classification	ANN	ACU: 0.91 Sensitivity: 100 Specificity: 82	No
Wu et al	Asia	2020	508	Risk/forecast	Classification	XGBoost	AUC: 0.76	No
Xin et al	Asia	2020	163	Diagnosis + Prognosis	Classification	XGBoost	AUC: 0.96 Sensitivity: 92	No
Yuan et al	Asia	2020	1090	Prognosis	Classification	Random forest	AUC: 0.88 Accuracy: 85	No
Daniel et al	Europe	2021	60	Prognosis	Classification	CNN	Accuracy: 99 Sensitivity: 93 Specificity: 99	No
Jeong et al	Asia	2021	586	Prognosis	Classification	Random forest	Sensitivity: 68	No
Krishnamurthy et al	Asia	2021	90,000	Risk/forecast	Classification	CNN	AUC: 0.95 Accuracy: 89 Sensitivity: 94 Specificity: 88	No
Ohara et al	Asia	2021	440	Therapy	Classification	RNN	Accuracy: 95	No
Parab et al	Asia	2021	57	Prognosis	Regression	ANN	MSE: 2.06	No
Peng et al	Asia	2021	198	Diagnosis	Regression	DNN	MSE: 11.62	No
Rashed-Al-Mahfuz et al	Asia	2021	400	Diagnosis	Classification	Random forest	AUC: 0.97 Accuracy: 97 Sensitivity: 96 Specificity: 99	No
Schena et al	Europe	2021	758	Diagnosis + Prognosis	Classification	ANN	Accuracy: 80	No
Senan et al	Asia	2021	400	Diagnosis	Classification	Random forest	Accuracy: 100 Sensitivity: 100	No
Shang et al	North America	2021	2350	Diagnosis	Classification	Ensemble model	Sensitivity: 87 Specificity: 97	No
Zhang et al	Asia	2021	115,344	Risk/forecast	Classification	ANN	AUC: 0.89	Yes

Most of the included articles (*n* = 51) were published from 2019 to 2021. Among the 68 articles selected for data extraction, the majority were published by authors from organizations based in Asia (*n* = 33; 48.5%). The remaining articles were published by authors from Europe (*n* = 17; 25%), North America (*n* = 12; 17.6%), Africa (*n* = 5; 7.35%) and South America (*n* = 1; 1.47%). The analyzed studies were classified as observational.

### Study aim

A total of 28 studies focused on the use of ML algorithms in disease prognosis analysis, 21 investigated the use of ML techniques on diagnosis (4 evaluated both), 12 evaluated the risk of developing the disease, and 3 investigated the use of ML in CKD treatment. Among the articles focusing on prognosis, the majority studied the application of ML in evaluating CKD progression (*n* = 13) and mortality (*n* = 8).

### Study populations and sample size

The most commonly investigated study population consisted of patients with CKD and healthy subjects (*n* = 26; 38.2%), followed by patients with CKD only (*n* = 16; 23.5%) and patients with CKD treated with hemodialysis (*n* = 12; 17.6%). The sample size investigated in the selected articles varied from a minimum of 30 individuals to a maximum of 550,000 (median = 776; IQR 400–12,020).

### Data sources

The majority of the included articles analyzed data obtained from single-hospital registries (*n* = 33; 48.5%), datasets provided by universities (*n* = 15; 22.1%), and datasets collected in multi-center studies (*n* = 12, 17.6%). Five studies analyzed health insurance data (7.35%) and 3 studies used data provided by national health services (4.41%).

The most commonly used data were various combinations of demographic data along with individual clinical characteristics and laboratory data (*n* = 60; 82.24%), followed by data obtained by medical imaging technologies (*n* = 5; 7.35%) and genomic data (*n* = 3; 4.41%).

### Models

The number of models tested and reported in each article varied from a minimum of 1 model to a maximum of 10 (mean = 3). The most frequently tested model class was tree algorithms (*n* = 58, 33.53%), such as random forest (*n* = 27, 15.61%), decision trees (*n* = 10, 5.78%) and extreme gradient boosting (*n* = 9, 5.20). Subsequently, neural networks (NNs) were often inspected (*n* = 44, 16.18%), especially the multilayer perceptron (MLP) (*n* = 28, 16.18%). Another popular choice of machine learning model class was Support Vector Machines (*n* = 25, 14.45%) and logistic regression (*n* = 18, 10.45%) with various regularizations. Another popular method that we did not classify into a larger model class was the non-parametric k-Nearest Neighbors algorithm (*n* = 8, 2.31%). The complete list of models can be found in Table 2.

**Table 2:** List of machine learning models used in the selected papers

Model class	Specific model	*n*		%	
Neural networks	Feedforward NN/multilayer perceptron (MLP)	44	28	25.43	16.18
	Convolutional NN (CNN)		9		5.20
	Recurrent NN and long short-term memory NN (RNN)		5		2.89
	Auto-encoder		1		0.58
	Extreme learning machine		1		0.58
Tree Algorithms	Random forest	58	27	33.53	15.61
	Decision trees		10		5.78
	Extreme gradient boosting (XGBoost)		9		5.20
	Gradient boosting machine		5		2.89
	Bagged decision trees		3		1.73
	Extremely randomized trees		2		1.16
	Light gradient boosting machine		1		0.58
	Adaptive boosting machine		1		0.58
	Categorical boost		1		0.58
Support Vector Machines	Support vector machines	25	22	14.45	12.72
	Genetic algorithm based on SVM		1		0.58
	Particle swarm optimization SVM		1		0.58
	Simulated annealing particle swarm optimization SVM		1		0.58
Logistic Regression	Logistic regression	18	13	10.45	7.51
	LASSO logistic regression		3		1.73
	Ridge logistic regression		1		0.58
	Elastic net logistic regression		1		0.58
Others	k-Nearest neighbors (kNN)	28	8	16.18	4.62
	Gaussian Naïve Bayes		4		2.31
	Ensemble model		3		1.73
	Linear regression		2		1.16
	(Adaptive) Neuro-fuzzy Inference System		2		1.16
	Partial Least Square Regression		1		0.58
	Hidden Markov Model (HMM)		1		0.58
	k-Means		1		0.58
	Cox regression		1		0.58
	Hierarchical clustering		1		0.58
	Genetic programming		1		0.58
	Linear discriminant analysis (LDA)		1		0.58
	Markov decision process (MDP)		1		0.58
	Hierarchical clustering		1		0.58

The models were also classified in larger model families to present a general overview. Some models that we were not able to classify in larger model families were classified as “Others”

All the articles implemented supervised learning algorithms, 57 (83.8%) of them addressed classification tasks and 11 (16.2%) regression tasks.

**Fig. 2:**
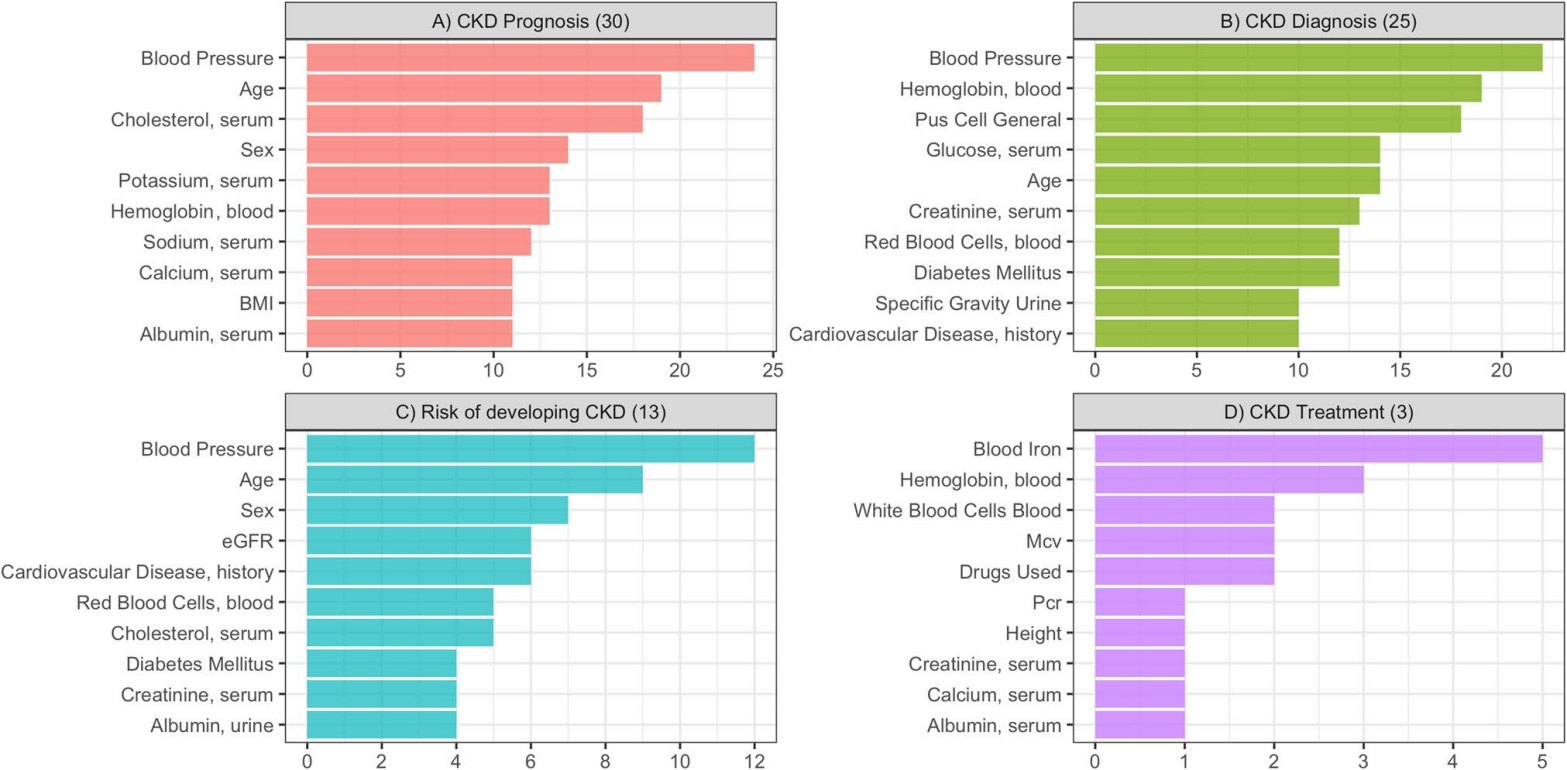
Occurrence of variables in the selected articles, divided per aim

The majority of the included articles (*n* = 52) specified the total number of features used to train the models. These models used a highly variable number of features, ranging from 4 to 6624 (median = 24; IQR = 17—46). Of the 68 included studies, 55 specified the variables used in the models (*n* = 130). The most frequently used features are reported in Fig. 2.

### Performance metrics

The most common performance metrics were accuracy (*n* = 30, 17.05%) and the area under the receiver operating characteristic curve (often also referred to as ROC-AUC, AUROC, AUC, or C-statistic) (*n* = 30, 17.05%). Subsequently, other classification metrics, such as sensitivity (*n* = 29, 16.48%), specificity (*n* = 24, 13.64%), precision (*n* = 16, 9.09%), and F1-score (*n* = 14, 7.95%) were often used to compare the machine learning models. Note that all the aforementioned metrics, except ROC AUC, were used for classification and required establishing a risk threshold as a decision boundary. ROC AUC conversely did not require setting a decision threshold as it was calculated by iterating over all the decision thresholds. In terms of regression, the most used metrics for comparison were mean absolute error (*n* = 6, 3.41%) and root mean squared error (*n* = 5, 2.84%). The full list of the metrics and how often they occurred can be found in Table 3.

**Table 3:** List of metrics and their occurrence in number and percentages of the selected papers

Name	*n*	%	Task
Accuracy	30	17.05	Classification
ROC AUC/C statistic	30	17.05	Classification
Sensitivity/recall	29	16.48	Classification
Specificity	24	13.64	Classification
Precision/positive predictive value (PPV)	16	9.09	Classification
F1 score	14	7.95	Classification
Matthews correlation coefficient	7	3.98	Classification
Mean absolute error (MAE)	6	3.41	Regression
Root mean squared error (RMSE)	5	2.84	Regression
Negative predictive value (NPV)	3	1.70	Classification
R2/coefficient of determination	3	1.70	Regression
Mean squared error (MSE)	2	1.14	Regression
Precision-recall AUC (AUPRC)	2	1.14	Regression
Bayesian information criterion (BIC)	1	0.57	Regression/classification
Cohen’s kappa statistic	1	0.57	Classification
Jaccard index/intersection over union	1	0.57	Classification
Normalized mean squared error (NMSE)	1	0.57	Regression
q2 Statistic	1	0.57	Regression

Furthermore, the last column specifies for which task the metric is used

### Best performing models, and their performances

In the included articles, neural networks were the models that commonly performed best (*n* = 28, 41.18%) compared to the median performance of other models, such as MLP (*n* = 18, 26.47%) and convolutional neural networks (*n* = 7, 24.53%). Tree-based algorithms performed best (*n* = 24, 35.29%); these algorithms included Random Forest (*n* = 16, 23.53%) and Extreme Gradient Boosting (*n* = 5, 7.35%). The results for Support Vector Machines (*n* = 5, 7.35%) were also noteworthy. A complete list of the best performing models in the selected papers can be found in Table 4.

**Table 4:** List of the best performing models throughout the selected papers, classified by model family

Model class	Model	*n*		%	
Neural networks	Feedforward NN/multilayer perceptron (MLP)	28	18	41.18	26.47
	Convolutional NN (CNN)		7		10.29
	Recurrent NN and long short-term memory NN (RNN)		3		4.41
Tree algorithms	Random forest	24	16	35.29	23.53
	Extreme gradient boosting (XGBoost)		5		7.35
	Decision tree		2		2.94
	Gradient boosting machine		1		1.47
Support vector machines	Support vector machines	5	5	7.35	7.35
Logistic regression	LASSO logistic regression	3	2	4.41	2.94
	Logistic regression		1		1.47
Others	Ensemble model	8	3	11.76	4.41
	k-Nearest Neighbors (kNN)		1		1.47
	Genetic algorithms		1		1.47
	Hierarchical clustering 1		1		1.47
	Hidden Markov Model		1		1.47
	Markov decision processes		1		1.47

In terms of performance, we compared the metrics of prediction models, diagnostic models and risk prediction models separately. Of the 25 (36.76%) machine learning models for diagnosis, 19 papers reported accuracy. Three models reported the highest accuracy of 1.00 while the lowest reported accuracy is 0.80 (mean = 0.95, median = 0.98). Sensitivity was reported 15 times, with a maximum of 1.00, a minimum of 0.56, a mean of 0.95 and a median of 0.99. In addition, specificity was reported in 13 cases (max = 1.00, min = 0.79, mean = 0.96, median = 0.99). The ROC-AUC was reported in 6 papers (max = 0.99, min = 0.91, mean = 0.941, median = 0.94).

For the prediction models (*n* = 32, 47.06%), 15 papers reported the ROC-AUC with a maximum of 0.96 and a minimum of 0.69 (mean = 0.82, median = 0.82). Ten papers reported accuracy, ranging from 0.54 to 0.99, with a mean of 0.85 and a median of 0.87. Sensitivity was reported 8 times, ranging from 0.54 to 0.93 (mean = 0.765, median = 0.76), and specificity was reported 5 times (max = 0.99, min = 0.78, mean = 0.917, median = 0.96).

Next, the risk prediction models (*n* = 12, 17.65%) showed ROC-AUC 9 times (max = 0.96, min = 0.76, mean = 0.864, median = 0.86) and accuracy 4 times (max = 0.99, min = 0.82, mean = 0.901, median = 0.91).

Finally, 3 (4.41%) papers focused on therapy, one of which reported an accuracy of 0.95, while the other two focused on outcome differences (*p*-values).

### Most common variables and most important ones

The total number of variables used in the included studies was 813. The five most common ones were: Blood Pressure (*n* = 62, 7.63%), Age (*n* = 45, 5.54%), Hemoglobin (*n* = 37, 4.55%), Creatinine (serum) (*n* = 31, 3.81%) and Sex (*n* = 31, 3.81%).

Nonetheless, to better capture how variables were used in the selected papers, we classified the variables into 4 subsets (CKD Prognosis, CKD Diagnosis, Risk of Developing CKD, CKD Treatment) based on the primary aim the authors stated their model would have attempted to achieve.

Regarding CKD Prognosis, 342 variables were used out of 813 total (42%). The most common ones were: Blood Pressure (*n* = 24, 7%), Age (*n* = 19, 5,56%), Cholesterol (serum) (*n* = 18, 5.26%), Sex (*n* = 14, 4%) and Hemoglobin (blood) (*n* = 13, 3.8%), with the most important variables being: Age, Hemoglobin and Proteinuria.

Concerning CKD Diagnosis, 311 variables were used out of 813 total (38.25%). The most common ones were: Blood Pressure (*n* = 22, 7%), Hemoglobin (blood) (*n* = 19, 6.1%), Pus Cell General—used to indicate the number of dead white cells in urine—(*n* = 18, 5.79%), Age (*n* = 14, 4.50%) and Glucose (serum) (*n* = 14, 4.50%). The most important variables in this case were Albumin, Creatinine, and Hemoglobin.

With regard to Risk of Developing CKD, 137 variables were used out of 813 total (16.85%). The most common ones were: Blood Pressure (*n* = 12, 8.75%), Age (*n* = 9, 6.57%), Sex (*n* = 7, 5.11%), History of Cardiovascular Disease (*n* = 6, 4.38%) and estimated Glomerular Filtration Rate (eGFR) (*n* = 6, 4.38%). The most important variables were Age, GFR and Blood Pressure.

Finally, regarding CKD Treatment, 23 variables were used out of 813 total (2.83%). The most common ones were: Blood Iron (*n* = 5, 21.74%), Hemoglobin (*n* = 3, 13%), Drugs Used (*n* = 2, 8.70%), MCV (*n* = 2, 8.70%) and White Blood Cells (blood) (*n* = 2, 8.70%). Regarding this aim, no weights were listed in the examined articles.

The complete spreadsheet with all variables and percentages can be found in Supplemental Material, together with the most important variables, divided per aim.

### Fairness

Other than using PROBAST to assess risk of bias, we also assessed fairness based on how the authors explicitly used variables. In some studies, variables were not fully listed, and in such cases, if the variable (sex, or race/ethnicity) was not indexed, we considered the feature as not included in the general model.

Out of 68 studies, 43 included gender in the model and 12 included race/ethnicity. When Non-Hispanic Whites were part of the assessed cohort, they were the majority group, ranging from 87 to 31%. Ten out of 68 studies addressed both gender and race/ethnicity, and included these variables in the model.

Race/ethnicity was included in 4 out of 12 studies predicting risk, in 5 out of 28 studies predicting prognosis, and in 3 out of 21 studies classifying diagnosis. It was never included in models investigating prognosis and diagnosis combined, and therapeutics.

### Clinical Deployment

Regarding Diagnosis, just one model was actually deployed in a clinical environment [60]. The authors applied a lasso regression with metabolites as features, achieving an accuracy of 99%; the authors used data from a real clinical context, and therefore they deployed and evaluated their model performance on a clinical context, nevertheless, they did not validate their model. Regarding Prognosis, just 3 studies were conducted in a clinical setting [49, 50, 62]. Komaru et al. [49] predicted 1-year mortality following the start of hemodialysis through hierarchical clustering and achieved an AUC of 0.8; the authors used data from a clinical prospective study to deploy and evaluate their model. Furthermore, they validated the used clusters. Kanda et al. [50] applied a support vector machine model onto a real population in an observational study to deploy and evaluate their model. The authors achieved an accuracy of 89% through 13 variables; unfortunately, they did not disclose the weights of the variables nor did they validate the model, and therefore we do not know which variables were the most important. Akbilgic et al. [62] used a model based on a Random Forest algorithm, and achieved an AUC of 0.69; the most important features were eGFR, Spontaneous Bacterial Peritonitis, Age, Diastolic Blood Pressure and BUN. The authors used data from a real clinical context to deploy and evaluate their model; furthermore, they validated their results and model internally. Regarding Risk of developing CKD, one study’s model was used in a clinical context [42]. The authors used a NN, achieving an AUC of 0.89, using retinal images as features from a clinical context to deploy, evaluate and validate their model. Finally, regarding CKD Treatment, one study’s model was used in a clinical environment [26]; they presented their results through differences in achieved values by their algorithms, and the best performance was achieved by a NN. They evaluated the model with clinical data, but did not validate it.

### Quality assessment

According to the PROBAST assessment tool [18], most of the included articles showed an overall low risk of bias (*n* = 48; 67.6%), and 65 (91.5%) of the included articles showed low applicability. Moreover, only 8.5% of the included studies scored less than 70% in the reporting guidelines for machine learning predictive models in biomedical research developed by Luo and colleagues [19]. The complete quality assessment can be found in Supplemental Material.

## Discussion

This systematic review describes how machine learning has been used for CKD. Six overarching themes were found, each of which underlines the need for further consideration by the scientific community.

First, despite the ever-growing number of studies focusing on the topic, a staggeringly low amount are being considered for actual clinical implementation. In this review, just 5 out of 68 articles tried to deploy their model in a real clinical setting. This might indicate either that the technology is not ready yet, or, considering 4 of these 5 articles were published in the last 3 years, that the technology is just starting to creep into real clinical settings. Recent evidence suggests that it is paramount to test newly developed algorithms in clinical settings before trying to deploy them [88]. Despite promising laboratory results, clinical translation is not always guaranteed. As an example, when studying the feasibility of providing an automated electronic alarm for acute kidney injury in different clinical settings, substantial heterogeneity in the findings among hospitals was described, with the worrying result of a significantly increased risk of death for some hospitals [89].

Second, as expected, the most important features were profoundly related to the main aim the authors were pursuing. In this regard, there were no surprises in the studied topics as the most important features were related to conditions known to lead to CKD diagnosis, worsening of prognosis and risk of developing CKD (e.g., age, comorbidities, systolic and diastolic blood pressure and eGFR values).

Third, a lack of consistency in reporting results was found. Most of the studies chose to report accuracy, but this was not the norm. Furthermore, while accuracy provides information on model performance, it fails to consider class imbalance and data representation. This is extremely important as accuracy in highly unbalanced datasets can be very high by always predicting the same binary outcome because of a flawed model. For instance, considering a low prevalence disease, if the algorithm is flawed for it always predicts a negative event, the accuracy will be high, but the veracity of the model will not [90]. As a result, AUCs and ROCs better measure the model precision without requiring the definition of a risk threshold. Twenty-nine authors chose to express their results including AUCs and ROCs: the minimum value was 0.69 and the maximum was 0.99 (mean: 0.83, median: 0.84). These results best express how precise the algorithms were and confirm the overall high performance of the assessed models.

Fourth, a common conundrum regarding feature selection and output was found in studies assessing CKD diagnosis. The definition of CKD requires certain variables to be present in order to make a diagnosis, thus including those variables in the model might be considered mandatory. Nonetheless, including those variables forces the model to streamline its decision process to a simple match in altered values, effectively transforming a complex machine learning model into a linear decision flow-chart, the performance of which will always be stellar.

This phenomenon is especially clear in four of the studies this systematic review assessed [36, 39, 46, 47]. In these studies, the same database [91] is used, and accuracy, sensitivity, specificity, and ROC-AUC are never below 98%. We believe researchers should carefully assess the variables used in their machine learning models to make sure that no data leakage is present between features and results.

Fifth, model bias and fairness were almost never considered. This is critical, as both biased and unfair models will not achieve the same results in different demographics, and their societal impact could exasperate disparities in certain populations. These issues need to be further explored before any model can be implemented at point of care.

Finally, among the included studies, only 6 evaluated their models in a clinical setting [26, 42, 49, 50, 60, 62], and only 3 were validated [42, 49, 62]. These studies showed promising results and did not report any unintended consequences after evaluation and/or validation. Notwithstanding the robust results described by the authors, as discussed before, recent evidence suggests that it is paramount to test newly developed algorithms in clinical settings to avoid adverse or unintended consequences [88, 89]. Taking into account the pinnacle of importance of validating ones’ results in real clinical contexts and not just “in lab”, in reading their results, their generalizability has to be questioned, especially since no multi-center validations were described among the validated models.

This systematic review presents a few limitations: first, only one database (PubMed) was used to collect studies of interest. It should be noted that systematic reviews are usually exhorted to use at least two databases as stated by the PRISMA statement. Nonetheless, as PubMed has grown to be one of the most used search engines for medical sciences this limitation should be self-amending. Secondly, this systematic review assessed only papers written in English since English is the most widely adopted and commonly used language for the publication of medical papers.

In addition to these limitations, due to this review’s design, all in vitro studies (on cellular substrates) were excluded. Consequently, the evidence presented in this review is not to be interpreted as definitive for all things concerning CKD, since in vitro studies (on cellular substrates), the insight of which is critical in understanding pathogenetic as well as therapeutic mechanisms, were not assessed.

Lastly, the majority of included studies did not evaluate the integration of ML models in daily clinical practice, therefore the results and discussion have to be considered largely from an academic standpoint. Despite these limitations, we feel this review advances the knowledge on the current state of data-driven algorithms to advance CKD diagnosis, prognosis and treatment.

Despite the potential benefits, the application of machine learning for CKD diagnosis, prognosis, and treatment presents several issues, namely fairness, model and result interpretability [90], and the lack of validated models. Result interpretability concerns reflect the inability to explain which aspects of the dataset used in the training phase led to a predicted result in a particular case [92, 93]. Therefore, as the trend in machine learning techniques moves from traditional algorithms (e.g., lasso regressions, support vector machine, and decision trees), to more complex ones (e.g., ensemble algorithms and deep learning), the interpretability concerns become more pronounced [90]. Notably, researchers highlighted the need for explainability and for models that could have a significant impact on patients' health [94, 95]. These models should be reported using best practice reporting guidelines such as the Transparent Reporting of a Multivariate Prediction Model for Individual Prognosis or Diagnosis (TRIPOD) [94] or MINimum Information for Medical AI Reporting (MINIMAR) [97]. Transparent and accurate reports are also fundamental in advancing multi-center validations of the applied models, which in turn is an essential step to ensure that only safe and sound models are applied on a large scale.

Most of the studies failed to report on the ethical issues revolving around their model development; the impact on the patient's well-being can also be affected by algorithmic bias [98, 99] and this can be worse in certain underrepresented populations. This concern is closely related to the generalizability of the developed model [100–102]. Specifically, retrospective data that are usually used during the training phase often have significant biases towards subgroups of individuals that have been defined by factors such as age, gender, educational level, socioeconomic status, and location [98]. The issues of fairness and bias in algorithms should be evaluated by investigating the models’ performance within population subgroups.

This systematic review underlines the potential benefits and pitfalls of ML in the diagnosis, prognosis, and management of CKD. We found that most of the studies included in this systematic review reported that ML offers invaluable help to clinicians allowing them to make informed decisions and provide better care to their patients; nonetheless most of those articles were not actually piloted in real life settings, and therefore, notwithstanding the excellent model performance results reported by authors, the technology might not be ready for mass real-time adoption or implementation.

Although future work is needed to address the viability, interpretability, generalizability, and fairness issues, to allow a safer translation of these models for use in daily clinical practice, the implementation of these techniques could further enhance the effective management of hospital resources in a timely and efficient manner by potentially identifying patients at high risk for adverse events and the need for additional resources.

We hope the summarized evidence from this article will facilitate implementation of ML approaches in the clinical practice.

## Supplementary Information

Below is the link to the electronic supplementary material.

Supplementary file1 (DOCX 22 KB)

Supplementary file2 (DOCX 40 KB)

Supplementary file3 (XLSX 15 KB)

Supplementary file4 (DOCX 10 KB)

## Data Availability

Data that support the findings of this study are available upon reasonable request from the corresponding author, AC.
