# Correction to: Predict, diagnose, and treat chronic kidney disease with machine learning: a systematic literature review

**DOI:** 10.1007/s40620-023-01609-9

**Published:** 2023-03-06

**Authors:** Francesco Sanmarchi, Claudio Fanconi, Davide Golinelli, Davide Gori, Tina Hernandez-Boussard, Angelo Capodici

**Affiliations:** 1 Department of Biomedical and Neuromotor Science, Alma Mater Studiorum, University of Bologna, Via San Giacomo 12, 40126 Bologna, Italy; 2 Department of Medicine (Biomedical Informatics), Stanford University, School of Medicine, Stanford, CA USA; 3 Department of Electrical Engineering and Information Technology, ETH Zurich, Zurich, Switzerland

**Correction to: Journal of Nephrology** 10.1007/s40620-023-01573-4

In abstract under Methods section the below sentence should not appear.

The review was registered on PROSPERO.

The original article has been corrected.

