# Supplementary material for: Predict, diagnose, and treat chronic kidney disease with machine learning: a systematic literature review
Source: J Nephrol. 2023 Feb 14;36(4):1101–17. doi: 10.1007/s40620-023-01573-4 (PMC10227138; doi:10.1007/s40620-023-01573-4)
Supplement: Supplementary file 2 — Supplementary file2 (DOCX 40 KB) [file 40620_2023_1573_MOESM2_ESM.docx]

**Supplementary material**

**Table S1. A Quality score of the included studies.**

| **Authors** | **Title** | **Abstract** | **Rationale** | **Objectives** | **Setting description** | **Problem definition** | **Data preparation** | **Build model** | **Report performance** | **Clinical implication** | **Limitations** | **Score (%)** |
| --- | --- | --- | --- | --- | --- | --- | --- | --- | --- | --- | --- | --- |
| Akl et al. | 1 | 0 | 1 | 1 | 0 | 1 | 1 | 1 | 0 | 1 | 0 | 64% |
| Kusiak et al. | 1 | 0 | 1 | 1 | 0 | 1 | 1 | 1 | 0 | 1 | 1 | 73% |
| Chen et al. | 1 | 1 | 0 | 1 | 1 | 1 | 1 | 0 | 1 | 1 | 0 | 73% |
| Luo et al. | 1 | 0 | 1 | 1 | 1 | 1 | 1 | 1 | 0 | 1 | 1 | 82% |
| Escandell-Montero et al. | 1 | 1 | 1 | 1 | 1 | 1 | 1 | 1 | 1 | 1 | 1 | 100% |
| Martínez-Martínez et al. | 1 | 0 | 1 | 1 | 1 | 1 | 1 | 1 | 1 | 1 | 0 | 82% |
| Barbieri et al. | 1 | 0 | 1 | 1 | 1 | 1 | 1 | 1 | 1 | 1 | 1 | 91% |
| Singh et al. | 1 | 0 | 1 | 1 | 1 | 1 | 1 | 1 | 1 | 1 | 1 | 91% |
| Barbieri et al. | 1 | 1 | 1 | 1 | 1 | 1 | 1 | 1 | 1 | 1 | 0 | 91% |
| Chen et al. | 1 | 1 | 1 | 1 | 1 | 1 | 1 | 0 | 1 | 1 | 0 | 82% |
| Norouzi et al. | 1 | 0 | 1 | 1 | 1 | 1 | 1 | 1 | 1 | 1 | 0 | 82% |
| Rodriguez et al. | 0 | 1 | 0 | 1 | 1 | 1 | 1 | 1 | 1 | 1 | 0 | 73% |
| Goldstein et al. | 0 | 0 | 1 | 1 | 0 | 1 | 1 | 1 | 1 | 1 | 1 | 73% |
| Polat et al. | 1 | 0 | 1 | 1 | 1 | 1 | 1 | 1 | 1 | 0 | 0 | 73% |
| Kleiman et al. | 1 | 0 | 1 | 1 | 1 | 1 | 1 | 1 | 1 | 1 | 1 | 91% |
| Kolachalama et al. | 0 | 0 | 1 | 1 | 1 | 1 | 1 | 1 | 1 | 1 | 1 | 82% |
| Tang et al. | 0 | 1 | 0 | 1 | 1 | 1 | 0 | 1 | 0 | 1 | 1 | 64% |
| Akbilgic et al. | 1 | 1 | 1 | 1 | 0 | 1 | 1 | **1** | 1 | 1 | 1 | 91% |
| Almansour et al. | 1 | 0 | 1 | 0 | 1 | 1 | 1 | 1 | 1 | 1 | 0 | 73% |
| Elhoseny et al. | 1 | 1 | 1 | 1 | 0 | 1 | 1 | 1 | 1 | 0 | 0 | 73% |
| Forné et al. | 1 | 1 | 1 | 1 | 1 | 1 | 1 | 1 | 1 | 1 | 1 | 100% |
| Galloway et al. | 1 | 1 | 1 | 1 | 1 | 1 | 1 | 1 | 1 | 1 | 0 | 91% |
| Guo et al. | 1 | 0 | 1 | 1 | 1 | 1 | 1 | 1 | 1 | 1 | 0 | 82% |
| Han et al. | 1 | 1 | 1 | 1 | 1 | 1 | 1 | 1 | 1 | 0 | 1 | 91% |
| Huang et al. | 1 | 1 | 1 | 1 | 0 | 1 | 1 | 1 | 1 | 0 | 0 | 73% |
| Kanda et al. | 1 | 0 | 0 | 1 | 1 | 1 | 0 | 1 | 0 | 1 | 1 | 64% |
| Kannan et al. | 1 | 1 | 1 | 1 | 0 | 1 | 1 | 1 | 1 | 1 | 0 | 82% |
| Kuo et al. | 1 | 1 | 1 | 1 | 1 | 1 | 1 | 1 | 1 | 1 | 1 | 100% |
| Lin et al. | 1 | 0 | 1 | 1 | 1 | 1 | 1 | 1 | 0 | 1 | 1 | 82% |
| Navaneeth et al. | 1 | 1 | 1 | 1 | 1 | 1 | 1 | 1 | 1 | 1 | 1 | 100% |
| Yu et al. | 1 | 0 | 1 | 1 | 1 | 1 | 1 | 1 | 1 | 1 | 1 | 91% |
| Aldhyani et al. | 1 | 0 | 1 | 1 | 1 | 1 | 1 | 1 | 1 | 0 | 0 | 73% |
| Belur Nagaraj et al. | 1 | 1 | 1 | 1 | 1 | 1 | 1 | 1 | 1 | 1 | 1 | 100% |
| Chen et al. | 1 | 0 | 1 | 1 | 0 | 1 | 1 | 1 | 1 | 1 | 1 | 82% |
| Dovgan et al. | 1 | 0 | 1 | 1 | 1 | 1 | 1 | 1 | 1 | 1 | 0 | 82% |
| Garcia-Montemayor et al. | 1 | 1 | 1 | 1 | 1 | 1 | 0 | 1 | 1 | 1 | 1 | 91% |
| Glazyrin et al. | 1 | 0 | 1 | 1 | 1 | 1 | 1 | 1 | 1 | 1 | 0 | 82% |
| Huang et al. | 0 | 1 | 1 | 1 | 1 | 1 | 1 | 1 | 1 | 1 | 1 | 91% |
| Inaguma et al. | 1 | 1 | 1 | 1 | 1 | 1 | 0 | 1 | 1 | 1 | 0 | 82% |
| Jeong et al. | 0 | 1 | 1 | 0 | 1 | 1 | 1 | 1 | 1 | 0 | 1 | 73% |
| Kanda et al. | 1 | 1 | 1 | 1 | 1 | 1 | 1 | 1 | 0 | 1 | 1 | 91% |
| Komaru et al. | 1 | 1 | 1 | 1 | 1 | 1 | 1 | 0 | 0 | 1 | 1 | 82% |
| Kumar et al. | 0 | 1 | 1 | 0 | 1 | 1 | 1 | 1 | 1 | 1 | 1 | 82% |
| Noh et al. | 1 | 1 | 1 | 1 | 1 | 1 | 1 | 0 | 1 | 1 | 0 | 82% |
| Nusinovici et al. | 0 | 1 | 1 | 1 | 1 | 1 | 1 | 1 | 1 | 1 | 1 | 91% |
| Ogunleye et al. | 0 | 1 | 1 | 1 | 0 | 1 | 1 | 1 | 1 | 1 | 1 | 82% |
| Pellicer-Valero et al. | 1 | 0 | 1 | 1 | 1 | 1 | 1 | 1 | 1 | 1 | 1 | 91% |
| Roth et al. | 1 | 1 | 1 | 1 | 1 | 1 | 1 | 0 | 1 | 1 | 1 | 91% |
| Sabanayagam et al. | 1 | 1 | 1 | 1 | 1 | 1 | 1 | 1 | 1 | 1 | 1 | 100% |
| Segal et al. | 1 | 1 | 1 | 1 | 1 | 1 | 0 | 1 | 1 | 1 | 0 | 82% |
| Shih et al. | 1 | 0 | 1 | 1 | 1 | 1 | 0 | **1** | 1 | 0 | 0 | 64% |
| Song et al. | 1 | 1 | 1 | 1 | 1 | 1 | 1 | 1 | 1 | 1 | 1 | 100% |
| Vitsios et al. | 0 | 0 | 1 | 1 | 1 | 1 | 1 | 1 | 0 | 1 | 0 | 64% |
| Weber et al. | 0 | 1 | 1 | 1 | 1 | 1 | 1 | 1 | 1 | 1 | 1 | 91% |
| Wu et al. | 1 | 1 | 1 | 1 | 1 | 1 | 0 | 0 | 1 | 1 | 1 | 82% |
| Xin et al. | 1 | 1 | 1 | 1 | 0 | 1 | 1 | 1 | 1 | 1 | 0 | 82% |
| Yuan et al. | 1 | 0 | 0 | 1 | 1 | 1 | 1 | 1 | 1 | 1 | 1 | 82% |
| Daniel et al. | 1 | 0 | 1 | 1 | 0 | 1 | 1 | 1 | 1 | 1 | 0 | 73% |
| Jeong et al. | 1 | 0 | 1 | 1 | 1 | 1 | 1 | 1 | 1 | 1 | 1 | 91% |
| Krishnamurthy et al. | 1 | 1 | 1 | 1 | 0 | 1 | 1 | 1 | 1 | 1 | 1 | 91% |
| Ohara et al. | 1 | 0 | 0 | 1 | 1 | 1 | 1 | 1 | 1 | 1 | 1 | 82% |
| Parab et al. | 1 | 1 | 1 | 1 | 1 | 1 | 1 | 1 | 1 | 1 | 1 | 100% |
| Peng et al. | 1 | 0 | 1 | 1 | 1 | 1 | 1 | 1 | 1 | 1 | 0 | 82% |
| Rashed-Al-Mahfuz et al. | 1 | 0 | 1 | 1 | 1 | 1 | 1 | 1 | 1 | 1 | 0 | 82% |
| Schena et al. | 1 | 1 | 1 | 1 | 1 | 1 | 1 | 1 | 1 | 1 | 1 | 100% |
| Senan et al. | 1 | 1 | 1 | 1 | 1 | 1 | 1 | 1 | 1 | 0 | 0 | 82% |
| Shang et al. | 0 | 0 | 1 | 1 | 1 | 1 | 1 | 1 | 1 | 1 | 1 | 82% |
| Zhang et al. | 1 | 1 | 1 | 1 | 1 | 1 | 1 | 1 | 1 | 1 | 1 | 100% |

**Table S1. B PROBAST checklist results.**

| **Authors** | **ROB participants** | **Applicability participants** | **ROB predictors** | **Applicability predictors** | **ROB outcomes** | **Applicability outcomes** | **ROB analysis** | **Overall ROB** | **Overall applicability** |
| --- | --- | --- | --- | --- | --- | --- | --- | --- | --- |
| Akl et al. | high concern | high concern | low concern | low concern | low concern | low concern | low concern | high concern | high concern |
| Kusiak et al. | low concern | low concern | low concern | low concern | low concern | low concern | low concern | low concern | low concern |
| Chen et al. | low concern | low concern | unclear concern | low concern | low concern | low concern | low concern | high concern | low concern |
| Luo et al. | low concern | low concern | low concern | low concern | low concern | low concern | low concern | low concern | low concern |
| Escandell-Montero et al. | low concern | low concern | unclear concern | low concern | low concern | low concern | low concern | high concern | low concern |
| Martínez-Martínez et al. | low concern | low concern | low concern | low concern | low concern | low concern | low concern | low concern | low concern |
| Barbieri et al. | low concern | low concern | low concern | low concern | low concern | low concern | low concern | low concern | low concern |
| Singh et al. | low concern | low concern | low concern | low concern | low concern | low concern | low concern | low concern | low concern |
| Barbieri et al. | low concern | low concern | low concern | low concern | low concern | low concern | low concern | low concern | low concern |
| Chen et al. | low concern | low concern | low concern | low concern | unclear concern | unclear concern | low concern | high concern | high concern |
| Norouzi et al. | low concern | low concern | low concern | low concern | low concern | low concern | low concern | low concern | low concern |
| Rodriguez et al. | low concern | low concern | low concern | low concern | low concern | low concern | low concern | low concern | low concern |
| Goldstein et al. | low concern | low concern | low concern | low concern | low concern | low concern | low concern | low concern | low concern |
| Polat et al. | low concern | low concern | low concern | low concern | low concern | low concern | unclear concern | high concern | low concern |
| Kleiman et al. | low concern | low concern | low concern | unclear concern | low concern | low concern | low concern | low concern | low concern |
| Kolachalama et al. | low concern | low concern | low concern | low concern | low concern | low concern | low concern | low concern | low concern |
| Tang et al. | low concern | low concern | low concern | low concern | low concern | low concern | low concern | low concern | low concern |
| Akbilgic et al. | high concern | high concern | low concern | low concern | low concern | low concern | low concern | high concern | high concern |
| Almansour et al. | low concern | low concern | low concern | low concern | low concern | low concern | low concern | low concern | low concern |
| Elhoseny et al. | low concern | low concern | low concern | low concern | low concern | low concern | low concern | low concern | low concern |
| Forné et al. | low concern | low concern | low concern | low concern | low concern | low concern | low concern | low concern | low concern |
| Galloway et al. | low concern | low concern | low concern | low concern | low concern | low concern | low concern | low concern | low concern |
| Guo et al. | low concern | low concern | low concern | low concern | low concern | low concern | low concern | low concern | low concern |
| Han et al. | low concern | low concern | low concern | low concern | low concern | low concern | low concern | low concern | low concern |
| Huang et al. | low concern | low concern | low concern | low concern | low concern | low concern | low concern | low concern | low concern |
| Kanda et al. | low concern | low concern | low concern | low concern | low concern | low concern | low concern | low concern | low concern |
| Kannan et al. | low concern | low concern | low concern | low concern | low concern | low concern | unclear concern | high concern | low concern |
| Kuo et al. | low concern | low concern | low concern | low concern | low concern | low concern | low concern | low concern | low concern |
| Lin et al. | low concern | low concern | unclear concern | unclear concern | low concern | low concern | low concern | high concern | high concern |
| Navaneeth et al. | low concern | low concern | low concern | low concern | low concern | low concern | low concern | low concern | low concern |
| Yu et al. | low concern | low concern | low concern | low concern | low concern | low concern | low concern | low concern | low concern |
| Aldhyani et al. | low concern | low concern | unclear concern | low concern | low concern | low concern | low concern | low concern | low concern |
| Belur Nagaraj et al. | low concern | low concern | low concern | low concern | low concern | low concern | low concern | low concern | low concern |
| Chen et al. | low concern | low concern | low concern | low concern | low concern | low concern | high concern | high concern | low concern |
| Dovgan et al. | low concern | low concern | unclear concern | low concern | low concern | low concern | low concern | high concern | low concern |
| Garcia-Montemayor et al. | low concern | low concern | low concern | low concern | low concern | low concern | low concern | low concern | low concern |
| Glazyrin et al. | low concern | low concern | unclear concern | low concern | low concern | low concern | unclear concern | high concern | low concern |
| Huang et al. | low concern | low concern | low concern | low concern | low concern | low concern | low concern | low concern | low concern |
| Inaguma et al. | low concern | low concern | low concern | low concern | low concern | low concern | low concern | low concern | low concern |
| Jeong et al. | low concern | low concern | unclear concern | unclear concern | low concern | low concern | low concern | high concern | high concern |
| Kanda et al. | low concern | low concern | low concern | low concern | low concern | low concern | high concern | high concern | low concern |
| Komaru et al. | low concern | low concern | low concern | low concern | low concern | low concern | unclear concern | high concern | low concern |
| Kumar et al. | low concern | low concern | low concern | low concern | low concern | low concern | low concern | low concern | low concern |
| Noh et al. | low concern | low concern | low concern | low concern | low concern | low concern | low concern | low concern | low concern |
| Nusinovici et al. | low concern | low concern | low concern | low concern | low concern | low concern | low concern | low concern | low concern |
| Ogunleye et al. | low concern | low concern | low concern | low concern | low concern | low concern | low concern | low concern | low concern |
| Pellicer-Valero et al. | low concern | low concern | low concern | low concern | low concern | low concern | low concern | low concern | low concern |
| Roth et al. | low concern | low concern | low concern | low concern | low concern | low concern | low concern | low concern | low concern |
| Sabanayagam et al. | low concern | low concern | low concern | low concern | low concern | low concern | low concern | low concern | low concern |
| Segal et al. | low concern | low concern | unclear concern | low concern | low concern | low concern | low concern | high concern | low concern |
| Shih et al. | low concern | low concern | low concern | low concern | low concern | low concern | low concern | low concern | low concern |
| Song et al. | low concern | low concern | low concern | low concern | low concern | low concern | low concern | low concern | low concern |
| Vitsios et al. | low concern | low concern | unclear concern | low concern | low concern | low concern | low concern | high concern | low concern |
| Weber et al. | low concern | low concern | low concern | low concern | low concern | low concern | low concern | low concern | low concern |
| Wu et al. | low concern | low concern | low concern | low concern | low concern | low concern | low concern | low concern | low concern |
| Xin et al. | low concern | low concern | unclear concern | low concern | low concern | low concern | low concern | high concern | low concern |
| Yuan et al. | low concern | low concern | unclear concern | low concern | low concern | low concern | unclear concern | high concern | low concern |
| Daniel et al. | low concern | low concern | low concern | low concern | low concern | low concern | low concern | low concern | low concern |
| Jeong et al. | low concern | low concern | unclear concern | low concern | low concern | low concern | low concern | high concern | low concern |
| Krishnamurthy et al. | low concern | low concern | low concern | low concern | low concern | low concern | low concern | low concern | low concern |
| Ohara et al. | low concern | low concern | unclear concern | low concern | low concern | low concern | low concern | high concern | low concern |
| Parab et al. | low concern | low concern | low concern | low concern | low concern | low concern | low concern | low concern | low concern |
| Peng et al. | low concern | low concern | low concern | low concern | low concern | low concern | low concern | low concern | low concern |
| Rashed-Al-Mahfuz et al. | low concern | low concern | low concern | low concern | low concern | low concern | low concern | low concern | low concern |
| Schena et al. | low concern | low concern | low concern | low concern | low concern | low concern | low concern | low concern | low concern |
| Senan et al. | low concern | low concern | low concern | low concern | low concern | low concern | unclear concern | high concern | low concern |
| Shang et al. | unclear concern | unclear concern | low concern | low concern | low concern | low concern | unclear concern | high concern | high concern |
| Zhang et al. | low concern | low concern | low concern | low concern | low concern | low concern | low concern | low concern | low concern |
