# Supplementary material for: Predict, diagnose, and treat chronic kidney disease with machine learning: a systematic literature review
Source: J Nephrol. 2023 Feb 14;36(4):1101–17. doi: 10.1007/s40620-023-01573-4 (PMC10227138; doi:10.1007/s40620-023-01573-4)
Supplement: Supplementary file 4 — Supplementary file4 (DOCX 10 KB) [file 40620_2023_1573_MOESM4_ESM.docx]

| **Main Aim** | **Most Important Features** |
| --- | --- |
| Diagnosis | Albumine (x2)  BMI  BUN  Calcium  Diastolic Blood Pressure  Dimethylargine  Gamma-Glutamyvaline  GFR  Hemoglobin (x2)  High density lipoprotein-colesterol (mmol/l)  Hydroxyasparagine  N-6-succinyladenosine  N-acetylanine  P/C ratio  PVC (x2)  Proteinuria (g/24 h)  Red blood cell count  Creatinine, Serum (x2)  Specific Gravity, urine  Underlying disease  Weight |
| Prognosis | Age (x4)  Albumine (x2)  Alkaline phospate  Anemia  Arterial pressure  BUN  Calcium (x2)  CKD stage  Creatinine  DBP  Diabetes  Diagnosis  Dialysis time (hours weekly)  Dialysis time (Total)  Dialysis Vintage  Direct Microscopy  Diuresis  eGFR (x2)  EPO (x2)  Hemoglobin (x4)  Hepatitis B Surface Ag  Hypertensive crisis events per year  Kt/V  Lipid-lowering drugs  osteopontin  PTH  pulse pressure  Recently diagnosed hypertension  SBP  Secondary Hyper-parathyroidism of Renal Origin  Sodium level  systolic blood pressure  Total Bilirubine serum  Total drug prescriptions per year  Troponine Serum  UACR  Ulcer of Lower Limb, Unspecified  Urine Protein (x5)  Weight (x3) |
| Risk of developing CKD | Age (x4)  Albumine to Creatine Ratio  Blood Pressure (x3)  BUN  Cardiovascular disease history  Chronic glomerulonephritis  Creatinine  Diabetes (x2)  Endothelin 1  Erythrocytes, Urinary  GFR (x4)  Glucose Fasting (x2)  Gout  HDL  Hypoglycemic drugs  Left atrium diameter  NAG enzyme level, Urinary  No presence of anal cancer (x2)  PC aa C38:0 (metabolite)  Protein and creatinine ratio, Urinary  Proteinuria  Sex  SM C18:1 (metabolite)  Smoking habit  Sulfonamides  Total cholesterol  Triglycerides  Urea nitrogen, blood  Uric acid |
